# Effect of Maxillary Expansion and Protraction in Class III Children on Quality of Life, Dentofacial and Upper Airway Characteristics: A Controlled Clinical Trial

**DOI:** 10.1111/ocr.12935

**Published:** 2025-04-18

**Authors:** Stjepan Spalj, Martina Zigante, Vedrana Tudor, Taner Öztürk, Ahmet Yağcı, Juan Martin Palomo

**Affiliations:** ^1^ Faculty of Dental Medicine, Department of Orthodontics University of Rijeka Rijeka Croatia; ^2^ Faculty of Dental Medicine and Health, Department of Dental Medicine J. J. Strossmayer University of Osijek Osijek Croatia; ^3^ Faculty of Medicine, Department of Family Medicine Universtiy of Rijeka Rijeka Croatia; ^4^ Faculty of Dentistry, Department of Orthodontics Erciyes University Kayseri Türkiye; ^5^ Department of Orthodontics, School of Dental Medicine Case Western Reserve University Cleveland USA

**Keywords:** airway management, interceptive orthodontics, maxillary expansion

## Abstract

**Objective:**

To explore the relationship between early dentofacial orthopaedic treatment, improvement in the width of oropharynx and nasopharynx, and quality of life.

**Materials and Methods:**

Thirty‐three prepubertal children with skeletal Class III (median age 9 years; 56% females) received treatment with a maxillary expander and facemask. These subjects were matched with two control groups: one comprising an equal number of untreated Class III individuals, and the other consisting of untreated Class I controls. Cephalograms were analysed, and both children and their parents self‐administered the Child Perceptions Questionnaire, Parental‐Caregiver Perceptions Questionnaire and Family Impact Scale.

**Results:**

Treated Class III cases showed significant increases in the nasopharyngeal and oropharyngeal airway width (*p* ≤ 0.033), with greater changes in the nasopharyngeal width compared to untreated Class III cases (*p* = 0.040). Compared to untreated Class III and Class I groups, treated Class III cases exhibited reduced mandibular prominence and sagittal skeletal Class, increased overjet, overbite, vertical facial dimension, and greater retroclination and retrusion of mandibular incisors (*p* ≤ 0.011). Prior to and following orthodontic treatment, Class III cases reported a lower quality of life across all dimensions compared to Class I controls (*p* ≤ 0.032). An increase in maxillary anterior movement and oropharyngeal width correlated with a decrease in functional limitations reported by children (*r* = −0.411‐(−0.413)); (*p* ≤ 0.022).

**Conclusion:**

Maxillary expansion and protraction in prepubertal Class III children can enhance upper airways width, and children associate these improvements with a reduction in functional limitations.

## 1 Introduction

Class III is relatively uncommon, occurring in about 6% of 12‐year‐old and 8% of 18‐year‐old Caucasian adolescents in Croatia, characterised by midface deficiency and/or excess in the mandible [1]. While a somewhat prominent chin can be seen as aesthetically pleasing in males, men with a concave profile tend to be less satisfied with their appearance compared to those with a flat or convex profile [2, 3].

Airways seem to be associated with sagittal malocclusions, particularly the size and position of the mandible. Class III mandibular protrusion is linked to a higher oropharyngeal volume and a larger posterior airway space at the tongue base compared to Class II mandibular retrusion [4, 5]. The literature remains inconclusive on whether breathing problems influence skeletal morphology or if skeletal morphology affects breathing problems. In Class III cases, the mandibular dental arch is broad but shortened, while the maxillary arch is narrow, making expansion a viable treatment option [6]. A meta‐analysis in 2018 synthesised data on pharyngeal airway changes after rapid maxillary expansion (RME) or facemask (FM) protraction in published studies in comparison to growth‐induced changes in untreated controls using two‐ or three‐dimensional assessments [7]. It concluded that an increase in the upper airway and nasal passage, but not the lower airway, can be achieved by maxillary expansion and/or protraction. However, it looks like maxillary advancement by FM without expansion does not affect the upper airway more than normal growth, nor does surgical maxillary advancement [8]. This most recent systematic review was based only on one paper for each of these two findings.

Minor differences were found in the upper and lower pharynx as a consequence of RME/FM treatment compared to normal growth. However, changes in cranial flexion are notable with the head tipping forward. This can be attributed to an increase in nasal cavity volume, some more increase in the upper pharynx and less decrease in the lower pharynx in treated subjects than controls, improvement in respiratory function, and consequent flexion of the head [9, 10].

The aim of this study was to explore how effective early orthopaedic treatment of Class III children is in terms of upper airways width and dentofacial changes when controlling for the effect of physiological growth, and also how treatment influences oral function and psychosocial impacts. The research question was formulated according to Population, Intervention, Comparison, Outcome and Study design (PICOS) strategy: (P) prepubertal Class III children; (I) maxillary expansion and protraction orthopaedic treatment; (C) untreated Class I and untreated Class III subjects; (O) changes in airway and dentofacial characteristics, and quality of life; (S) controlled clinical trial. Hypotheses were that the maxillary expansion/protraction treatment stimulates growth in the maxilla and nasopharyngeal airways width when compared to the growth in untreated Class III and Class I children. Additionally, it was expected that children with Class III have poorer quality of life than those with Class I, but orthopaedic treatment improves the quality of life, reaching that of Class I children.

## 2 Materials and Methods

Thirty‐three consecutive subjects with skeletal Class III (median age 9 years, interquartile range 8–9 years; 56% females) referred to the University Dental Clinic Rijeka, Croatia were enrolled. Inclusion criteria were Wits ≤ 0.5 mm, prepubertal growth stage CS ≤ 2 [11]. Exclusion criteria were patients with congenital airway stenosis, syndromes, cleft lip and/or palate, systemic diseases influencing facial growth, and mental disability. Recruitment began in February 2018, lasted 5 years (it stopped in 2020 due to COVID pandemic) and the study completed in April 2023.

The acrylic expander was bonded in the maxillary arch (from deciduous canine to the first permanent molar) followed by hyrax screw activation twice per day during the 3 weeks. The FM was then applied on hooks embedded in acrylic hyrax (medium elastics, direction 45° down). Instructions were given to wear FM 14–16 h per day. The expander was removed approximately 9 months after cementing and a maxillary acrylic plate with a bimaxillary labial bow (horizontal wire passing on the equator of mandibular incisors) was given for retention. Lateral cephalograms were taken before treatment and 1 month after expander removal.

The treated group was matched with the same number of untreated Class III and Class I cases whose cephalograms were selected from the American Association of Orthodontics Legacy Collection growth studies (Bolton‐Brush, Michigan, Forsythe, Oregon, Denver, and Burlington in the United States) and Erciyes University in Türkiye. Untreated controls were selected in August 2024. The cephalometric variables used are presented in Table S1. Thirty cephalograms were analysed twice in 1‐week intervals and pointed to great to excellent reliability (intraclass correlation coefficient ranging from 0.790–0.992).

Instruments Child Perceptions Questionnaire, Parental‐Caregiver Perceptions Questionnaire, and Family Impact Scale were self‐administered by children and their parents in the Class III group before and 1 month after treatment [12, 13, 14]. Additionally, 33 orthodontically untreated children presenting Class I normal occlusion or mild malocclusion (grade I of the Index of Orthodontic Treatment Need—Dental Health Component), with the absence of cavities and gingivitis, and their parents also administered the instruments [15]. Those children were selected from local elementary schools during the preventive programme and were matched by age and gender to the treated Class III group. Instruments assessed aspects of functional limitations (FL), oral symptoms (OS), emotional well‐being (EW), and social well‐being (SW) related to oral conditions. Parents/caregivers also reported FL, OS, EW and SW observed in their children. Additionally, parents/caregivers reported aspects of parental emotions (PE), family activities (FA), family conflicts (FC), and financial burdens (FB) related to oral conditions.

The primary outcomes were width of oropharynx and nasopharynx, while quality of life and dentofacial characteristics were secondary outcomes. There were no changes to methods and trial outcomes after the trial commenced. No harm or unintended effects were seen.

The sample size was calculated with a control of type I error of 0.05 and a power of 0.80 using an online tool [16]. If the expected difference in the change of nasopharyngeal width induced by treatment is 3.83 ± 2.8 mm and the effect of growth is 0.67 ± 1.98, at least 11 participants are needed in each group [17].

The normality of data was checked by the Kolmogorov‐Smirnov test. For data that had a normal distribution, parametric statistics were used (analysis of variance and Student–Newman–Keuls post hoc test for independent samples and *t*‐test for pairs), while if the distribution was not normal, non‐parametric tests (Kruskal‐Wallis with Mann–Whitney post hoc test with Bonferroni correction for independent samples and Wilcoxon test for paired samples). Spearman correlation was applied to test the association between changes in variables (ΔT2‐T1). Discriminant analysis was employed to identify the changes in dentofacial and airway characteristics that differentiate treated and untreated groups. Commercial statistical software SPSS 22 was used (IBM SPSS, Armonk, US).

## 3 Results

Basic demographic and clinical characteristics are presented in Table 1. Class I controls mostly differed from Class III cases (untreated and treated). Class III untreated cases had more mandibular incisors retroclination, higher facial height, and shorter midface and mandibular length than treated Class III cases. Post hoc analyses did not find significant differences in the nasopharyngeal airway width between groups.

**TABLE 1:** Basic demographic and clinical characteristics.

Variable	Class III untreated (*N* = 33)	Class III treated (*N* = 33)	Class I untreated (*N* = 33)	*p* ^1^
Gender (number of females in sample)	12	14	13	0.881
Age (median and IQR*)	9 (8–10)	9 (8–9)	9 (8–9)	0.881
Maturation stage; CS	1 (1–2)	1 (1–1)	1 (1–1)	0.098
OB	0.3 (−1.2–2.5)^a^	−0.1 (−0.5–1.8)^a^	2.5 (2.1–4.2)^b^	**< 0.001**
OJ	−1.3 (−2.7‐(−0.2))^a^	−1.0 (−1.9‐(−0.1))^a^	3.2 (2.8–3.8)^b^	**< 0.001**
SNA	79.7 (77.4–81.9)	80.8 (78.0–82.7)	81.7 (78.5–83.5)	0.205
SNB	79.7 (76.7–82.7)^a^	79.9 (77.7–82.6)^a^	76.8 (75.8–80.3)^b^	**0.001**
ANB	−0.4 (−1.7–0.8)^a^	−0.1 (−0.8–1.8)^a^	4.0 (2.4–4.6)^b^	**< 0.001**
Wits	−4.4 (−6.7‐(−3.2))^a^	−4.7 (−5.9‐(−2.9))^a^	−1.2 (−2.5–0.2)^b^	**< 0.001**
NAPg	0.0 (−5.0–2.8)^a^	−0.9 (−3.9–2.8)^a^	7.1 (3.2–8.9)^b^	**< 0.001**
CoA	71.1 (68.1–73.6)^a^	75.5 (74.1–78.7)^b^	73.7 (71.5–76.6)^b^	**< 0.001**
CoGn	96.5 (93.1–98.5)^a^	100.0 (96.0–103.7)^b^	91.8 (89.4–95.2)^c^	**< 0.001**
SN:ANS‐PNS	5.0 (0.9–9.4)^a^	0.7 (−1.3–2.1)^b^	0.1 (−1.0–2.5)^b^	**< 0.001**
ANS‐PNS:MeGo	26.3 (22.6–29.8)	23.4 (20.9–27.1)	23.8 (20.9–27.3)	0.273
Bjork	395.6 (392.3–400.1)^a^	391.7 (389.1–394.3)^b^	392.7 (389.5–394.8)^b^	**0.002**
Y axis	62.7 (60.6–67.0)^a^	64.7 (61.7–66.3)^a^	67.1 (64.0–68.3)^b^	**0.003**
CoGoMe	127.1 (122.2–130.1)	125.1 (122.1–128.9)	124.8 (119.7–127.2)	0.118
U1:ANS‐PNS	108.2 (104.1–113.1)	113.2 (106.0–117.3)	109.3 (107.3–114.4)	0.190
U1‐NA	1.9 (−0.1–3.2)	3.0 (1.2–3.8)	2.2 (1.0–3.5)	0.310
L1‐MeGo	88.8 (83.9–93.0)^a^	93.8 (89.4–96.5)^b^	98.2 (95.2–101.1)^c^	**< 0.001**
L1‐NB	3.1 (1.6–4.4)	3.3 (2.6–4.9)	3.6 (2.9–4.6)	0.225
Nasopharyngeal airway width	10.2 (7.5–10.7)	7.6 (6.0–9.5)	7.8 (5.6–10.6)	**0.038**
Oropharyngeal airway width	9.4 (7.3–12.7)	11.6 (8.7–13.2)	8.7 (7.5–11.5)	0.077
Hy:MeGo	8.1 (5.8–13.0)^a^	10.5 (5.6–13.5)^a^	6.1 (2.8–9.1)^b^	**0.002**
G'SnPg'	169.7 (167.7–175.7)^a^	171.3 (164.9–175.9)^a^	164.8 (162.9–179.2)^b^	**0.001**
Ls‐E	−2.9 (−5.2‐(−0.9))^a^	−4.1 (−6.3‐(−2.5))^a^	−1.5 (−3.0‐(−0.4))^b^	**< 0.001**
Li‐E	0.3 (−1.2–2.1)	−0.9 (−3.0–1.1)	0.1 (−1.0–0.6)	0.077

*Note:* Groups that share the same superscript letters do not differ significantly. Bold values indicates significance levels.

* Interquartile range.

^1^ Level of statistical significance.

**FIGURE 1:**
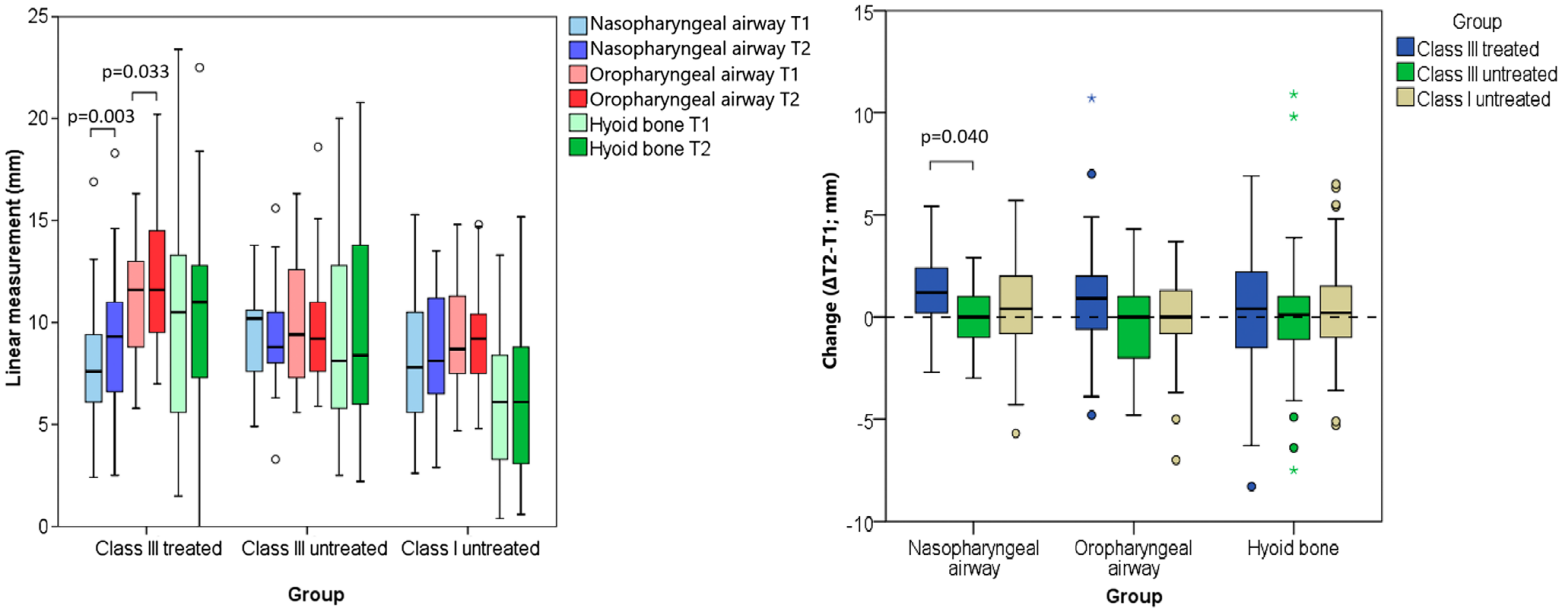
Comparison of airway dimension before and after 1 year in treated Class III subjects and untreated controls of Class III and Class I (left) and changes in upper airways width between groups (right). Circles represent outliers and asterisk represents extreme value. Parentheses connect groups that significantly differ.

Orthopaedic treatment induced an increase in the nasopharyngeal width (*p* = 0.003) and oropharyngeal width (*p* = 0.033; Figure 1). It also induced an increase in the distance of the hyoid bone from the mandibular base, which did not reach a level of statistical significance. No significant changes were seen in upper airways width in growing untreated Class III subjects nor untreated Class I subjects.

**FIGURE 2:**
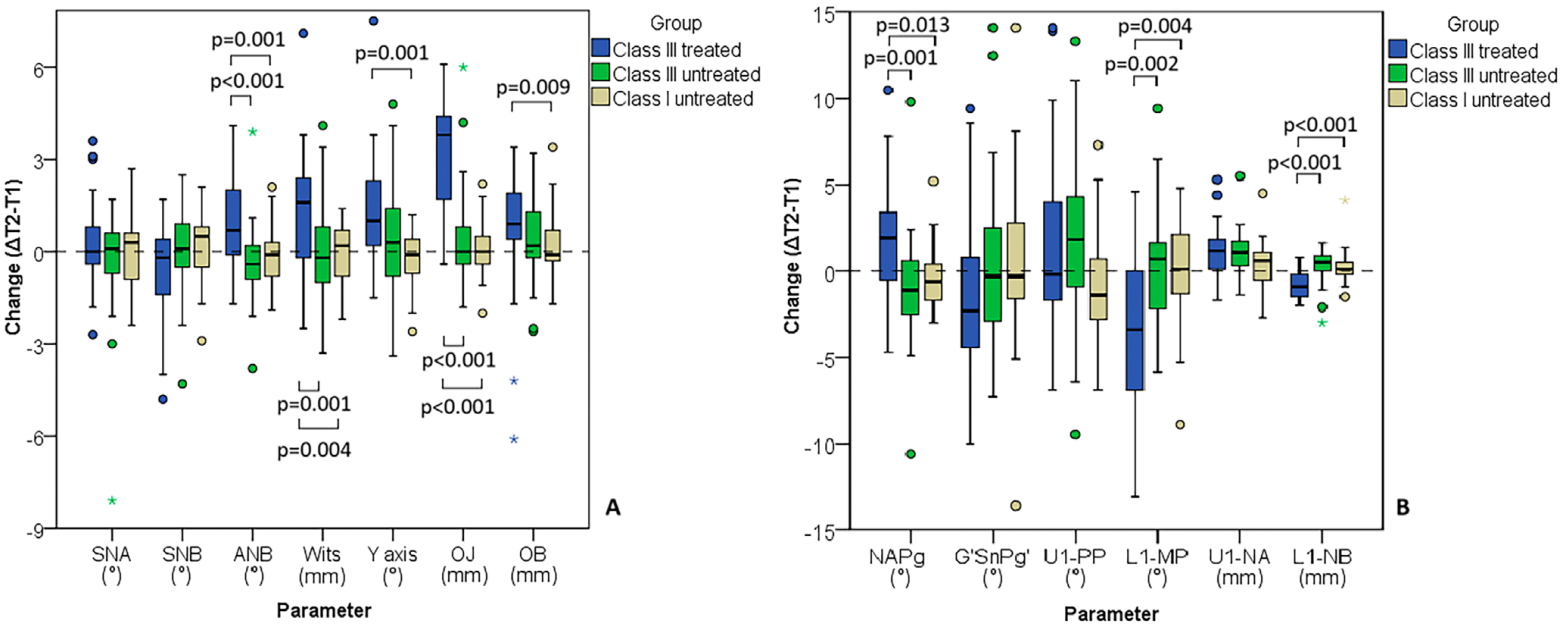
Comparison of changes in sagittal and vertical dimensions, incisors position, and facial profile between groups. Circles present outliers and asterisk indicates extreme value. Parentheses connect groups that significantly differ.

The extent of changes (ΔT2‐T1) in nasopharyngeal width differed between groups (*p* = 0.045), with the treated Class III cases showing greater changes compared to the untreated Class III controls (*p* = 0.040; Figure 1). No significant changes were detected in oropharyngeal width and hyoid bone position between groups. Groups differed in ANB, Wits, NAPg, Y axis, OB, OJ, L1:MeGo, and L1:NB (*p* ≤ 0.011) with Class III treated cases having a bigger change than untreated Class III, and in some parameters than Class I (Figure 2A,B). Treated Class III cases experienced an increase in OJ, OB, ANB, Wits, NAPg, CoA, CoGn, Y axis angle, U1:NA, while a reduction in L1:MeGo and L1:NB (*p* ≤ 0.023). Growing untreated Class I had an increase in CoA, CoGn, and U1:NA (*p* ≤ 0.041).

Changes in nasopharyngeal and oropharyngeal widths were not interrelated, while the hyoid bone position was linearly positively correlated to the oropharyngeal airway (*r* = 0.280; *p* = 0.005).

**FIGURE 3:**
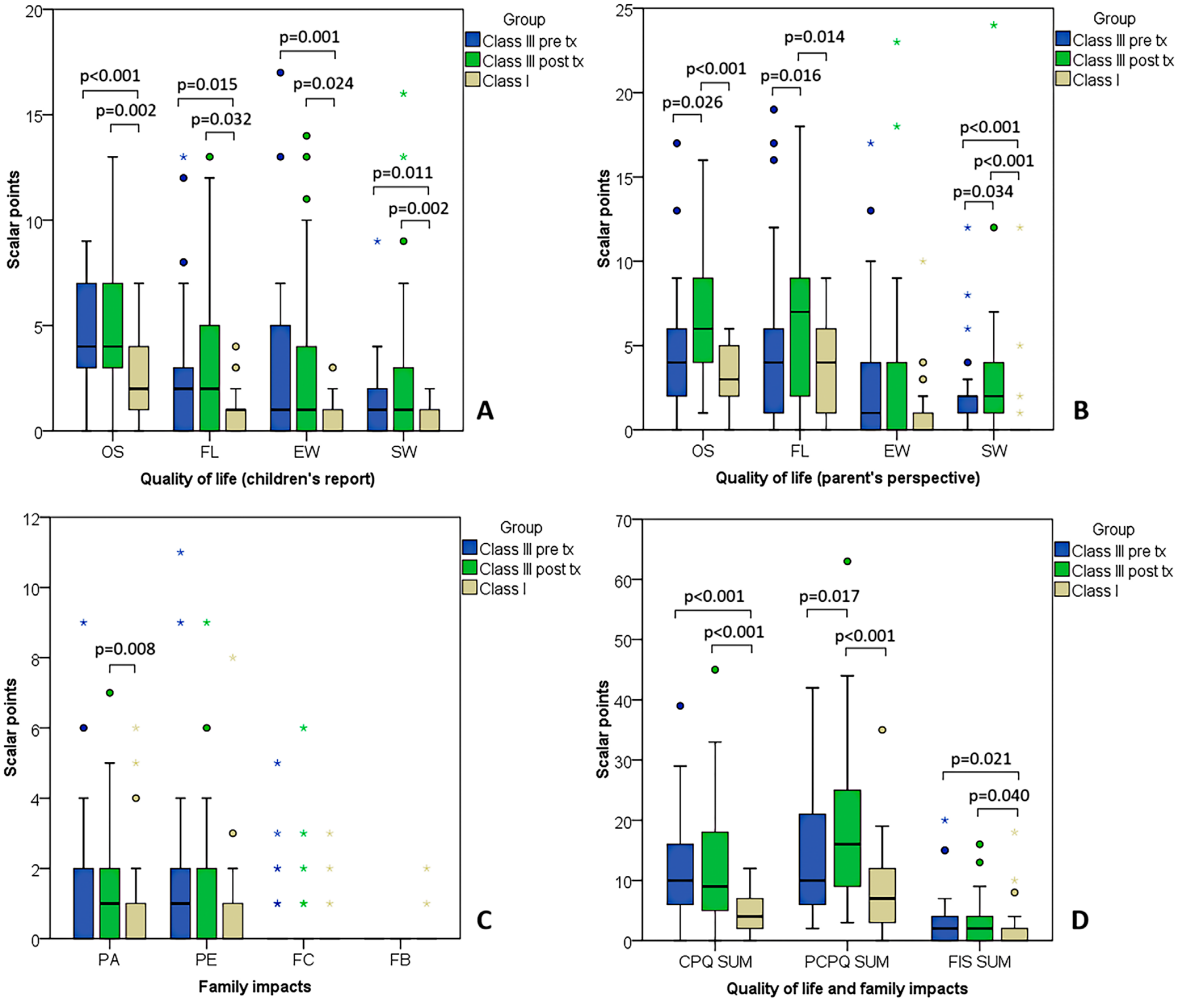
Comparison of quality of life reported by children and their parents between Class III (pre and post‐treatment) with Class I controls. Circles present outliers and asterisks extreme values. Parentheses connect groups that significantly differ.

Orthopaedic treatment did not significantly influence the quality of life reported by children. Class III children reported greater impairment in all quality of life dimensions than Class I controls, both before and after orthodontic treatment (*p* ≤ 0.016; Table 2; Figure 3A,D). Parents reported deterioration in children's OS, FL, and SW (*p* ≤ 0.026; Figure 3B).

**TABLE 2:** Comparison of quality of life in Class III cases before treatment and Class I untreated controls.

Variable	Class III before treatment (*N* = 33)	Class I untreated (*N* = 33)	*p* ^b^
OS (median (IQR^a^))	4 (3–8)	2 (1–4)	**< 0.001**
FL	2 (0–4)	1 (0–1)	**0.015**
EW	1 (0–5)	0 (0–1)	**0.001**
SW	1 (0–3)	0 (0–1)	**0.011**
OS parent	4 (2–6)	3 (2–5)	0.050
FL parent	4 (1–7)	4 (1–6)	0.811
EW parent	1 (0–5)	0 (0–2)	**0.040** ^c^
SW parent	2 (1–3)	0 (0–0)	**< 0.001**
PA	0 (0–2)	0 (0–0)	0.094
PE	1 (0–2)	0 (0–1)	0.058
FC	0 (0–1)	0 (0–0)	0.109
FB	0 (0–0)	0 (0–0)	0.154
CPQ SUM	10 (6–16)	4 (2–7)	**< 0.001**
PCPQ SUM	10 (6–22)	7 (3–13)	**0.028** ^c^
FIS SUM	2 (0–5)	0 (0–2)	**0.021**

^a^ Interquartile range.

^b^ Level of statistical significance.

^c^ Not significant when Bonferroni correction of *p* value for multiple comparisons with Class III after treatment is used.

Additionally, parents of Class III children rated the quality of life of their children even after treatment as more impaired than parents of Class I children (*p* < 0.001), particularly children's OS, FL and SW (*p* ≤ 0.014). Family impacts were higher in Class III children before treatment than in Class I (*p* = 0.021). After treatment, impacts were still higher (*p* = 0.040) particularly PA (*p* = 0.008) (Figure 3C,D). Changes in FL induced by orthopaedic treatment reported by children correlated with changes in the oropharyngeal airway (*r* = −0.413; *p* = 0.021) and SNA (*r* = −0.411; *p* = 0.022). Change in SW was the only one that correlated between children and parents (*r* = 0.384; *p* = 0.033). Changes in clinical variables were not related to changes in family impacts.

**FIGURE 4:**
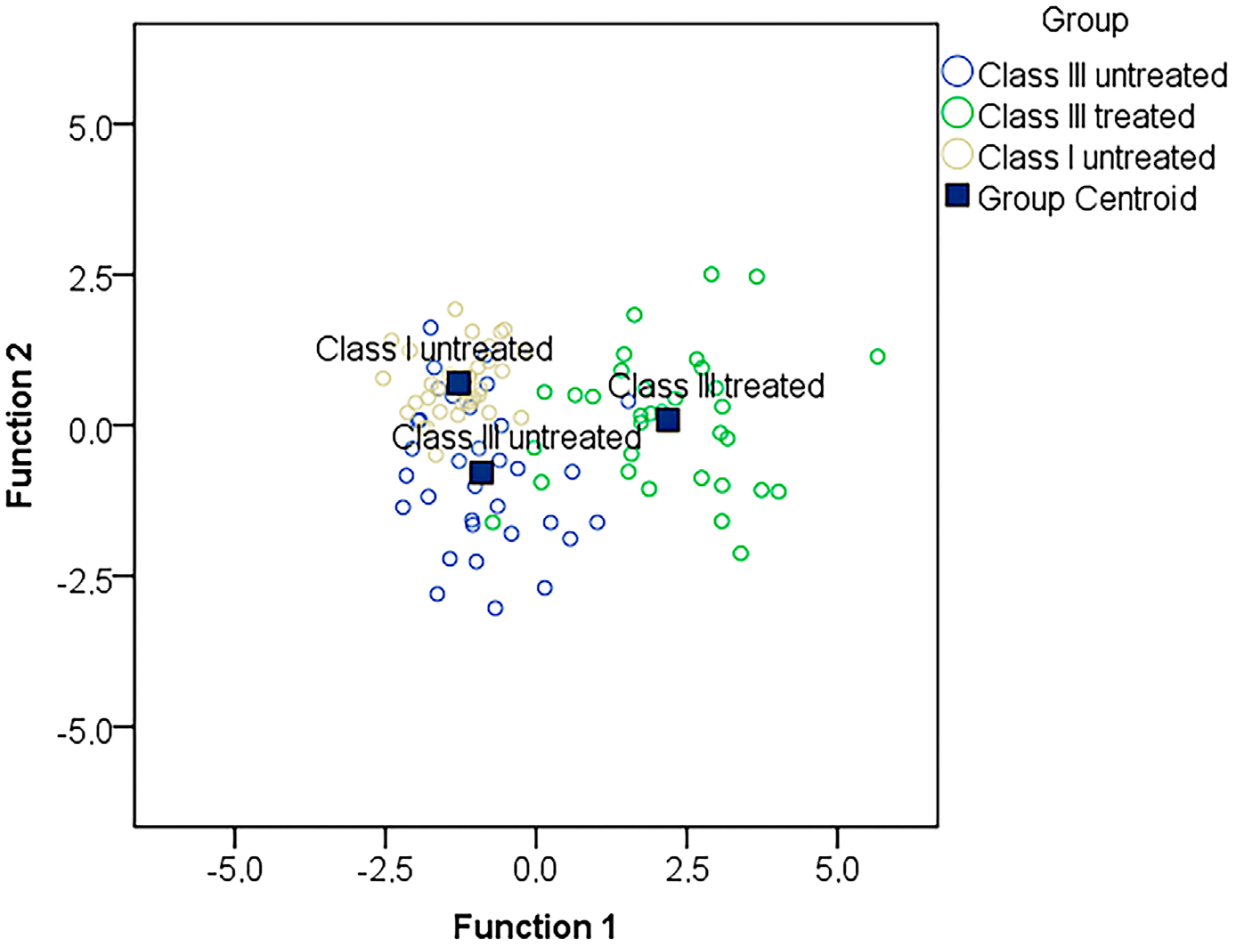
All groups scatter plot of the discriminant analysis.

The discriminant analysis detected only one statistically significant canonical function that accounted for 87.2% of the variability (*p* < 0.001). Graphically, the first function is shown on the X‐axis of Figure 3 and consists of the variables shown in Table S2. Figure 4 shows a clear difference along the X‐axis between treated Class III children and untreated children. Treated Class III children had the biggest increase in OJ, ANB, Wits, CoA, CoGn, Y axis, NAPg, oropharyngeal and nasopharyngeal airway, and OB while a decrease in L1:NA, L1:MeGo, SNB, Gl'SnPg’, Ls‐E while untreated Class I controls the least. Class III controls are in those characteristics more similar to Class I controls. The model correctly classifies 81% of cases, the most Class III treated (91%), and less Class I untreated (88%) and Class III untreated (64%).

## 4 Discussion

The present research determined that some airway improvements resulting from early orthopaedic treatment for Class III malocclusions are due to the treatment itself, not just growth. Additionally, children associate these treatment outcomes with enhancement in certain aspects of their daily functioning.

A significant portion of the effects of RME/FM treatment in this study was due to the clockwise rotation of the mandible, which increased the vertical dimension of the lower face and reduced the concave facial profile. The retroclination and retrusion of the mandibular incisors led to an increase in OJ. The posterior rotation of the mandible limited its forward growth displacement, resulting in less improvement in the oropharyngeal airway compared to the nasopharyngeal airway. Additionally, the retrusion and retroclination of the mandibular incisors caused the tongue to move backward, which did not favour the oropharyngeal airway.

Most of our data are in line with a Cochrane systematic review and a meta‐analysis which, in addition, pointed forward displacement of the maxilla as a treatment effect of FM [18, 19]. RME/FM is the most used protocol in Class III, considered a gold standard, implying that early treatment can reduce the need for surgical correction after growth seizes, or at least reduce the extent of surgery [19]. Recently published meta‐analyses brought interesting information. More maxillary protraction can be achieved in the short term when FM is combined with RME, and there is a gradual decrease of achieved anteroposterior sagittal maxillary advancement in the long term [20]. Therefore, a decrease in skeletal effects achieved in our study can also be expected.

When focusing on airways, there is no benefit of RME with protraction in Class III children when compared to only protraction [21]. FM treatment has a similar dentoskeletal outcome irrespective of whether it is provided in the late‐mixed or early‐permanent dentition stage or the early‐mixed dentition stage. So even if we enter into the pubertal growth phase, as some of our subjects entered during treatment, a similar outcome is achieved [22].

Comparing different RME protocols in children revealed that both expansion‐only (RME/FM) and alternate expansion and contraction (Alt‐RAMEC/FM) produce similar amounts of changes in the nasal cavity, nasopharyngeal, oropharyngeal airway, and maxillary sinuses, but Alt‐RAMEC/FM shifts the maxilla more forward and separates the circumaxillary sutures [23, 24]. Still, RME/FM used also in our study continues to be regarded as the gold standard.

Some other treatment approaches, such as the Frankel appliance, have been reported to increase upper airway dimensions. However, without a control group, the effects of normal growth are not accounted for [25]. This study's advantage lies in its inclusion of a control group, ensuring the effect of growth is controlled.

This study used lateral cephalograms which are routine in orthodontics, and the majority of assessments were based on 2D for a long time. The analysis of airways was therefore limited to measurements of the width of the oropharynx and nasopharynx. New technologies introduced possibilities for 3D analyses including volumes. Repeatability and reliability for linear measurements are similar in 2D and 3D with high correlation between 3D and 2D measurements [26]. CBCT did not become a standard in orthodontics.

Smaller children are also more prone to tonsillitis, which influences the assessment of nasopharyngeal dimension, which may add to heterogeneity in treatment changes reported in the current study. Also, staying still while taking images, proper positioning of the tongue, breathing, and swallowing are more difficult to control in children. Volumes assessment, assessed by CBCT, appears not to be an ideal representation of real conditions since they depend on the patient's head motion and movement [27, 28].

Class III children had a worse quality of life in the present study than their Class I peers. The relation between malocclusions and poorer quality of life in children has been reported previously [29]. However, it seems that all levels of malocclusion affected the oral FL and social lives, while only very severe malocclusion could develop OS and affect emotions and the overall oral health‐related quality of life (OHRQoL) [30]. No major changes in psychosocial and functional aspects of their life were reported by treated Class III children in our study. So even after treatment and clinically confirmed improvement in orofacial morphology, they did not reach the OHRQoL of Class I mates. Probably at a prepubertal stage, they can not appraise properly changes in facial appearance and relate it to orofacial function. Surely not enough questions were related to breeding and questionnaires were administered too soon after appliance removal (1 month). Maybe more time is needed for children to notice change. Still, they related the change in oropharyngeal width and maxillary anterior movement to a decrease in FL. Others reported that OS and FL worsen when starting orthodontic treatment, but OHRQoL improves after 6 months of treatment and also 1 month after appliance removal [31, 32].

Children and their parents provided different responses regarding the impact of orthopaedic treatment on OHRQoL. While children generally reported an improvement (though not statistically significant), parents tended to report a decline. This suggests that parents may not accurately assess their children's feelings, a finding consistent with previous reports [33]. The discrepancy may be because the instruments were primarily administered by mothers, who are likely more attuned to the bulkiness of the appliances used. They might have perceived the treatment as overly aggressive, with the actual changes not being as dramatic as they had anticipated.

Class III malocclusion influences family, especially parental activities when compared to Class I children. This is rather logical, as it takes some time and effort for parents to find an orthodontist, make an appointment, and reschedule their obligations to make consultation, diagnostics, and treatment.

According to others, maxillary protraction does not have psychosocial benefits, but it reduces the perceived need for orthognathic surgery [34, 35]. Contrary to orthopaedic treatment, surgical treatment of Class III strongly improves the quality of life of patients, even more than that of Class II subjects [36].

Discriminant analysis in our study confirmed the univariate analysis findings, summarising that treated Class III cases are distinct from both untreated groups. Notably, changes in upper airways width and stimulation of maxillary sagittal growth were not the most prominent differences between groups. The primary distinctions were the retroclination and retrusion of mandibular incisors and the increase in vertical dimension.

Some clinical characteristics of the untreated skeletal Class III group were more pronounced than those in the treated skeletal Class III group, yet they have not received any treatment, which does not comply with ethical requirements. Monitoring Class III children through multiple x‐rays without initiating treatment would be ethically questionable in modern practice. Therefore, using a historical cohort was the most appropriate approach. Additionally, untreated Class III cases are rare in available growth studies, so this sample was compiled by reviewing data from approximately a dozen such studies. The historical cohort could be considered a limitation; however, there is evidence that secular trends in the past 60 years did not influence the facial growth rate and growth direction much, so the historical cohort can be considered a valuable source of controls [37].

Additionally, the long‐term effects of treatment were not explored, which could provide more reliable conclusions. The ethical justification for retaking X‐rays of subjects, when not necessary for a new therapy, is highly questionable. Using a Wits value ≤ 0.5 mm as an inclusion criterion for skeletal Class III could be considered a limitation. However, the Wits value is influenced by other skeletal factors. Both Wits and ANB have drawbacks, but the inconsistency between those two assessments is mostly in high‐angle cases, which were not included in this sample [38]. A limitation is the lack of control of confounding factors, such as psychological state, family background, and support which could influence quality‐of‐life reporting and potentially result in biased outcomes. It has been confirmed that children experiencing lower self‐esteem, higher anxiety, and depression tend to report poorer quality of life [39]. Higher family income and parental education levels, and positive parenting practices are associated with reporting better quality of life [40].

In addition, other factors may affect airway and quality of life, such as family genetic history and past medical history. These may involve neuromuscular activity, alterations in smooth muscle tone (either increased or decreased), mucosal or smooth muscle hypertrophy, oedema, and encrusted secretions.

The advantage of the present study is the control of the effect of the growth using two control groups—untreated Class III and Class I cases. In addition, OHRQoL dimensions and family impacts were directly related to the amount of changes induced by treatment and compared to subjects with normal occlusion. The clinical implication might be that while orthodontic treatment can enhance orofacial morphology, it does not completely restore the quality of life to that of children without malocclusion. Future studies should include a bigger sample to enhance the generalisability of the results and the precision of statistical analysis, volumetric analysis, and connect changes to respiratory functional parameters and patient‐reported outcomes related to breathing.

## 5 Conclusion

Maxillary expansion and protraction in prepubertal Class III children can enhance upper airways width, facial profile, and overjet primarily through dentoalveolar changes rather than skeletal ones. These children typically experience a lower quality of life compared to their Class I counterparts, and orthopaedic treatment alone does not improve their quality of life enough to match that of Class I children. Improvements in airways width are associated with reduced functional limitations as perceived by the children.

## Conflicts of Interest

The authors declare no conflicts of interest.

## Supporting information


Data S1.

## Data Availability

Data are available on request from the authors.
