# Supplementary material for: Effect of Maxillary Expansion and Protraction in Class III Children on Quality of Life, Dentofacial and Upper Airway Characteristics: A Controlled Clinical Trial
Source: Orthod Craniofac Res. 2025 Apr 18;29(3):481–9. doi: 10.1111/ocr.12935 (PMC13139985; doi:10.1111/ocr.12935)
Supplement: Supplementary file 1 — Data S1. [file OCR-29-481-s001.docx]

Supplementary Table 1. Cephalometric variables assessed from lateral cephalograms

| Name of variable | Abbreviation / description |
| --- | --- |
| Sagittal dimension |  |
| Maxillary prognathism angle | SNA (°) |
| Mandibular prognathism angle | SNB (°) |
| Sagittal skeletal class angle | ANB (°) |
| Sagittal skeletal class appraisal | Wits (mm) |
| Skeletal facial convexity angle | NAPg (°) |
| Vertical dimension |  |
| Facial skeletal Y axis angle | NSGn (°) |
| Bjork polygon | Sum of NSAr, SArGo and ArGoMe (°) |
| Inclination of maxilla to anterior cranial base | SN:ANS-PNS (°) |
| Intermaxillary angle | ANS-PNS:MeGo (°) |
| Airways |  |
| Nasopharyngeal airway width | Shortest distance from the soft palate to the posterior pharyngeal wall (the point where the palatal plane intersects the pharyngeal wall) |
| Oropharyngeal airway width | Distance from the point where the tongue and mandible meet to the posterior pharyngeal wall |
| Distance of hyoid bone to mandibular plane | Hy:MeGo (mm) |
| Soft tissue |  |
| Facial convexity | Gl’SnPg’ (°) |
| Upper lip prominence | LsPrnPg’ (mm) |
| Lower lip prominence | LiPrnPg’ (mm) |
| Dentition |  |
| Overjet | OJ (mm) |
| Overbite | OB (mm) |
| Inclination of the most prominent maxillary incisor to the palatal plane | U1:ANS-PNS (°) |
| Inclination of the most prominent mandibular incisor to mandibular plane | L1:MeGo (°) |
| Protrusion of the most prominent maxillary incisor to the NA line | U1:NA (mm) |
| Protrusion of the most prominent mandibular incisor to the NB line | L1:NB (mm) |

Supplementary Table 2. Structural matrix of canonical functions. Correlations within groups between discriminant variables and standardized canonical discriminant functions are presented and variables are ordered by absolute magnitude of correlation within the function.

| Variable | Function | |
| --- | --- | --- |
| Δ OJ | 0.687^†^ | -0.052 |
| Δ L1:NA | -0.388^†^ | -0.148 |
| Δ ANB | 0.345^†^ | 0.186 |
| Δ L1:MeGo | -0.329^†^ | -0.136 |
| Δ Wits | 0.302^†^ | 0.113 |
| Δ NAPg | 0.275^†^ | 0.202 |
| Δ Y axis | 0.263^†^ | -0.213 |
| Δ SNB | -0.217^†^ | 0.031 |
| Δ CoA | 0.208^†^ | 0.195 |
| Δ CoGn | 0.207^†^ | 0.071 |
| Δ oropharyngeal airway | 0.186^†^ | 0.114 |
| Δ nasopharyngeal airway | 0.164^†^ | 0.154 |
| Δ G’SnPg’ | -0.137^†^ | 0.023 |
| Δ OB | 0.108^†^ | -0.098 |
| Δ Ls-E | -0.075^†^ | -0.059 |
| Δ U1:ANS-PNS | 0.071 | -0.373^†^ |
| Δ U1:NA | 0.119 | -0.339^†^ |
| Δ Li-E | -0.089 | 0.323^†^ |
| Δ CoGoMe | -0.046 | 0.278^†^ |
| Δ Bjork | 0.126 | 0.188^†^ |
| Δ SNA | 0.084 | 0.184^†^ |
| Δ Hy:MeGo | -0.009 | 0.088^†^ |

^†^Highest absolute correlation between each variable and any discriminant function.
